# Early Repolarization Syndrome: Diagnostic and Therapeutic Approach

**DOI:** 10.3389/fcvm.2018.00169

**Published:** 2018-11-27

**Authors:** Felix Bourier, Arnaud Denis, Ghassen Cheniti, Anna Lam, Konstantinos Vlachos, Masateru Takigawa, Takeshi Kitamura, Antonio Frontera, Josselin Duchateau, Thomas Pambrun, Nicolas Klotz, Nicolas Derval, Frédéric Sacher, Pierre Jais, Michel Haissaguerre, Mélèze Hocini

**Affiliations:** ^1^Electrophysiology and Ablation Unit, Bordeaux University Hospital (CHU), Pessac, France; ^2^Electrophysiology and Heart Modeling Institute, IHU Liryc, Pessac, France; ^3^Centre de Recherche Cardio-thoracique de Bordeaux, U1045, Université de Bordeaux, Bordeaux, France

**Keywords:** early repolarization syndrome, sudden cardiac death, J wave, ICD implantation, idiopathic ventricular fibrillation

## Abstract

An early repolarization pattern can be observed in 1% up to 13% of the overall population. Whereas, this pattern was associated with a benign outcome for many years, several more recent studies demonstrated an association between early repolarization and sudden cardiac death, so-called early repolarization syndrome. In early repolarization syndrome patients, current imbalances between epi- and endo-cardial layers result in dispersion of de- and repolarization. As a consequence, J waves or ST segment elevations can be observed on these patients' surface ECGs as manifestations of those current imbalances. Whereas, an early repolarization pattern is relatively frequently found on surface ECGs in the overall population, the majority of individuals presenting with an early repolarization pattern will remain asymptomatic and the isolated presence of an early repolarization pattern does not require further intervention. The mismatch between frequently found early repolarization patterns in the overall population, low incidences of sudden cardiac deaths related to early repolarization syndrome, but fatal, grave consequences in affected patients remains a clinical challenge. More precise tools for risk stratification and identification of this minority of patients, who will experience events, remain a clinical need. This review summarizes the epidemiologic, pathophysiologic and diagnostic background and presents therapeutic options of early repolarization syndrome.

## Introduction

Up to 10% of all sudden cardiac deaths are caused by primary electrical disorders or ion channel diseases. As consequence, the identification of genetic mutations affecting these ion channels has opened a new area of translational research in cardiac electrophysiology (1, 2). Over the last decades, an early repolarization pattern had been considered as a benign finding, it is frequently observed on surface ECGs, characterized by J-point and ST segment elevation in 2 or more contiguous leads. More recently, the early repolarization pattern has increasingly attracted attention as it has been reported as a risk to idiopathic ventricular fibrillation and sudden cardiac death in case-control studies, characterized as early repolarization syndrome. This review provides a historic, epidemiologic and pathophysiologic background and describes diagnostic and therapeutic approaches in the treatment of early repolarization syndrome.

## Historic perspective

In 1936, Shipley and Hallaran firstly described an early ST segment elevation in ECGs which they derived in a population of 200 young, healthy individuals. They observed this phenomenon in 25% of male and in 16% of female study participants (3). Two years later, in 1938, similar findings were observed in the surface ECG of an individual who died from hypothermia (4). In 1951, the term “Early Repolarization” was firstly used by Grant in a study on vector electrocardiography (5). Osborn described the classic J-wave in experimental hypothermia in 1953, presumably resulting from an increased dispersion of repolarization caused by a disproportionate abbreviation of the epicardial action potential compared to the endocardium (6).

Historically, early repolarization was described as a normal variant phenotype. Since the past decade, growing evidence exists about the association between an early repolarization phenotype and the incidence of sudden cardiac death (7). In 2008, Haïssaguerre et al. reported a significantly increased prevalence of early repolarization in patient cohort with history of idiopathic ventricular fibrillation (8).

## Epidemiology

Early repolarization can be observed in 1% up to 13% of the overall population, with a higher incidence in the more recent studies and in populations of athletes and adolescents. For a long time, early repolarization was associated with benign outcome (9–13). Recently, an association between early repolarization in the inferolateral leads and sudden cardiac death has been described in several studies. There may be two potential reasons for the observed variation in incidence of early repolarization between single studies: Firstly, the definition and interpretation of early repolarization used in different studies varies. Secondly, there are significant differences in baseline characteristics of studied populations. Studies which showed a correlation of early repolarization and sudden cardiac death included mainly Caucasian and Asian and less African or American individuals. It is noticeable that among all studied patients showing early repolarization, 75% were males (13–15). J point elevation is found more frequently in patients with idiopathic ventricular fibrillation than in healthy individuals (11). In concordance, in sudden cardiac deaths related to early repolarization, male gender is found in 75% (8, 13).

In a case-control study of Haïssaguerre et al. the prevalence of early repolarization was documented in 31% of patients with a history of idiopathic ventricular fibrillation, whereas only in 5% of healthy controls (8). In a meta-analysis, the absolute risk for sudden cardiac death in individuals with early repolarization syndrome is estimated to be 0.07% (16).

## Pathophysiology and genetics

**Figure 1:**
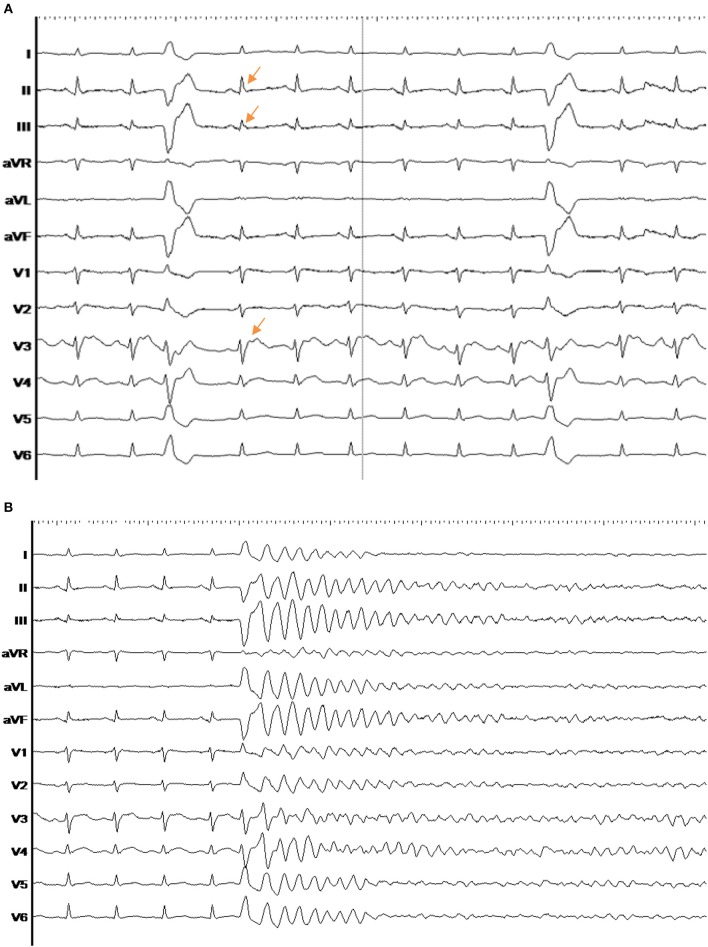
**(A)** ECG example of a patient Nr. 1 with early repolarization syndrome. Two frequent clinical PVCs, arising from the septal basal RV, are documented. **(B)** The clinical PVC of early repolarization syndrome patient Nr. 1 induces an episode of ventricular fibrillation.

In early repolarization syndrome patients, current imbalances between epi- and endo-cardial layers result in a dispersion of de- and repolarization. These imbalances manifest as J wave or ST segment elevation on the surface ECG. In the epicardium, a larger transient-outward K^+^ (I_to_) and Adenosine triphosphate-sensitive current (I_KATP_), and a reduced inward sodium (I_Na_) and inward calcium (I_CaL_) current than in the endocardium result in greater net repolarizing outward current flow during the early phase of the myocardial action potential. In early repolarization patients, a further increase in epicardial net outward current results in an increase of differences of action potential between epi- and endo-cardium. The resulting prominent notch in the action potential of ventricular epicardium but not endocardium induces a transmural voltage gradient during ventricular activation and manifests as J wave on the surface ECG. The electrical heterogeneity results in so-called phase 2 reentries, which produce closely coupled PVCs capable of initiating circus movement reentry and ventricular fibrillation. (Figures 1A,B, 3A,B). The amplitude of the J waves, representing a disequilibrium between epi- and endo-cardial currents, increases during bradycardia phases and vagotonia, short-long sequences are then more likely to trigger ventricular fibrillation.

Experimental studies showed that testosterone increases outward potassium currents, including the rapidly activating and the slowly activating component of the delayed rectifier potassium current, and decreased the inward L-type calcium current (17, 18). As the maintenance of the action potential dome is determined by a precise balance of currents, any agents that increase outward currents or decrease inward currents may increase the magnitude of the action potential notch, increasing the voltage gradient across the endo- and epi-cardium, thus augmenting ST-segment and J-point elevation.

The previously described male predominance in the prevalence of an early repolarization ECG pattern (13–15) may be attributable to the higher testosterone levels of males, resulting in an increased outward potassium current and an increased J point elevation.

Early repolarization is more frequently found in genetic relatives of patients with a history of arrhythmic sudden cardiac death, what suggests pro-arrhythmic genetic mutations and in several recent studies, early repolarization syndrome has been described as a kind of heritable disease. Population-based studies suggested some degree of inheritance of early repolarization ECG patterns (19–21). However, only a few genes have related to early repolarization syndrome have been identified by now. Mutations of KCNJ8, which encodes a subunit of the ATP-sensitive potassium channel, of the L-type calcium channel genes (CACNA1C, CACNB2B, CACNA2D1) as well as loss-of-function mutations of SCN5A are related to early repolarization and idiopathic VF (22–25). Most of the early repolarization syndrome causing gene mutations were detected in the sporadic cases and the current understanding of the genetic basis of early repolarization syndrome remains limited. It has recently been proposed that early repolarization syndrome may be a near-Mendelian or oligogenic inheritance disease (21).

## Diagnosis

An “early repolarization pattern” is diagnosed on the surface ECG as a sharp, positive deflection at the onset of the ST segment, following immediately after a positive QRS complex. Also, as an increased J point level may be hidden in the terminal QRS complex, early repolarization may be indicated by a slurring of the terminal QRS complex. A J point elevation, exceeding 0.1 mV, has to be present in two or more contiguous inferior and/or lateral leads. The onset of the QRS slurring has to be entirely above the ECG baseline level, and the angle between the tangents of the slurring and the initial R downslope has to be >10° (26). An “early repolarization syndrome” can be diagnosed, if the early repolarization pattern is found in a patient with a history of idiopathic ventricular fibrillation or polymorphic ventricular tachycardia. It is important to emphasize that an “early repolarization pattern” on its own is not a cardiac arrhythmic disease. The 2013 Expert Consensus Statement by Heart Rhythm Society (HRS), the European Heart Rhythm Association (EHRA), and the Asia Pacific Heart Rhythm Society (APHRS) summarizes diagnostic criteria: (27)

- Early repolarization syndrome **is diagnosed** by the presence of J-point elevation ≥1 mm in ≥2 contiguous inferior and/or lateral leads of a standard 12-lead ECG in a patient resuscitated from otherwise unexplained ventricular fibrillation/ polymorphic ventricular tachycardia.
- Early repolarization syndrome **can be diagnosed** in a SCD victim with a negative autopsy and medical chart review with a previous ECG demonstrating J-point elevation ≥1 mm in ≥2 contiguous inferior and/or lateral leads of a standard 12-lead ECG.
- Early repolarization pattern **can be diagnosed** by the presence of J-point elevation ≥1 mm in ≥2 contiguous inferior and/or lateral leads of a standard 12-lead ECG.

As an early repolarization pattern can be relatively frequently observed in the overall population, but also growing evidence exists that early repolarization is linked to sudden cardiac death, there is a specific need for risk stratification.

Life-threatening arrhythmias are often the first, unexpected clinical manifestation of early repolarization syndrome. An increase of J wave/ST segment amplitude has been described before the onset of ventricular fibrillation in early repolarization patients. Ventricular fibrillation is often initiated by short-long-short QRS sequences, with a single PVC falling into the T-wave of the preceding QRS complex (28).

**Figure 2:**
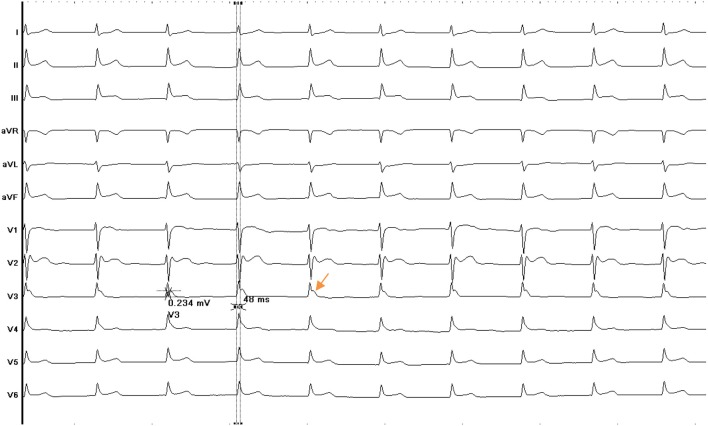
ECG of patient Nr. 2 presenting with early repolarization syndrome. J point elevation is predominantly present in lead v3.

**Figure 3:**
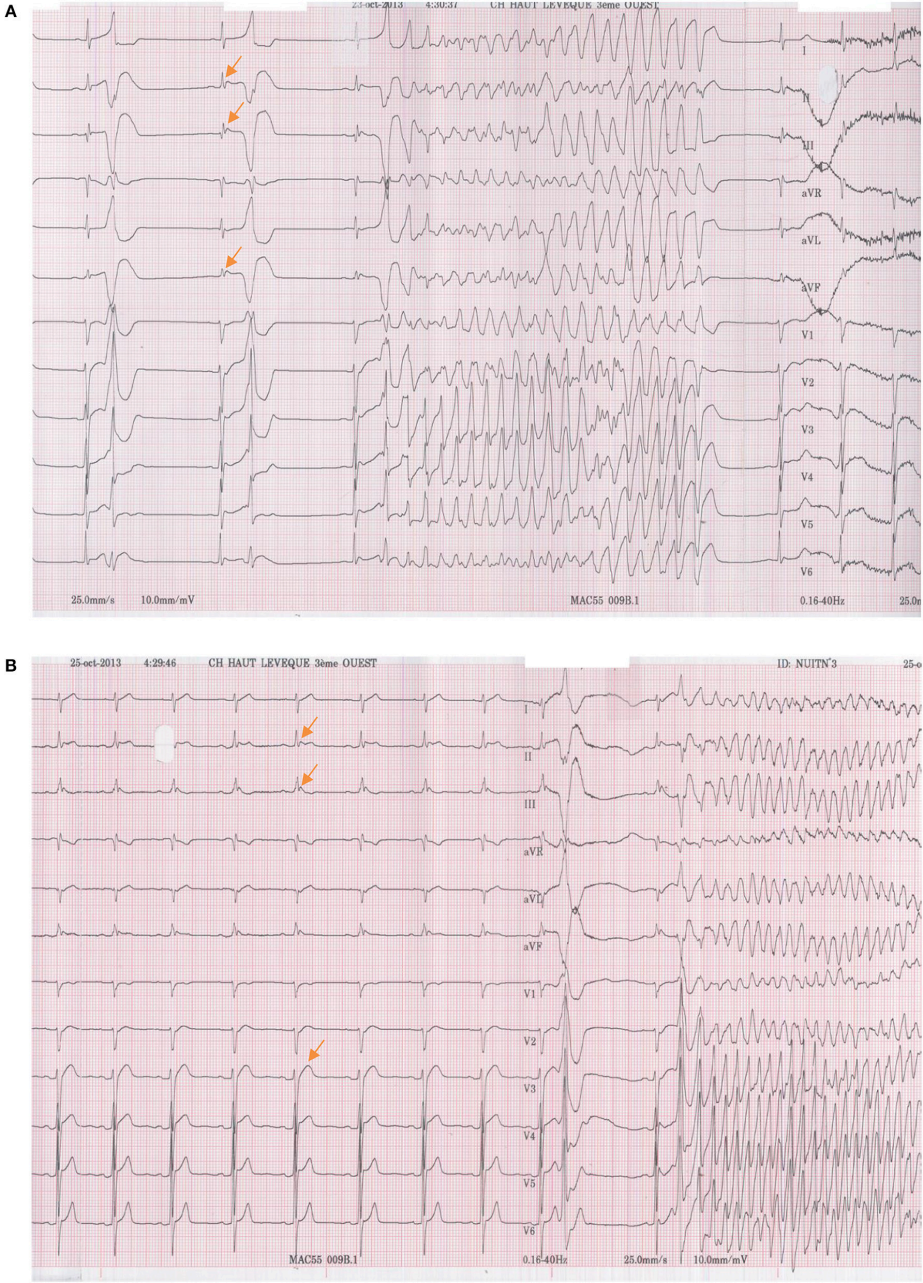
**(A)** An episode of non-sustained ventricular fibrillation, which occurred during baseline ECG recording of early repolarization syndrome patient Nr. 3. The VF episode was induced by the patient's early clinical PVC. **(B)** ECG recording of patient Nr. 3. The clinical PVC now induces an episode of sustained ventricular fibrillation.

**Figure 4:**
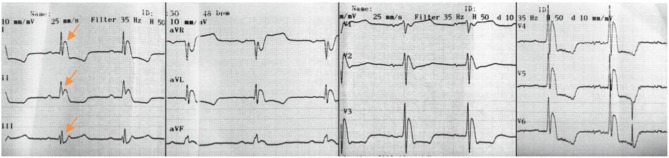
ECG recording of patient Nr. 4 shows a predominant early repolarization pattern in the inferior leads.

ECG examples of early repolarization patients are presented in Figures 1–4.

## Risk stratification

Based on different studies linking early repolarization pattern to the incidence of sudden cardiac death, the majority of patients will remain asymptomatic, while arrhythmic events and sudden cardiac death will only occur in few patients. Thus, more precise tools for risk stratification and the identification of this minority of patients, who will experience events, remain a clinical need and challenge.

Observational studies subcategorized early repolarization ECG patterns to distinguish benign and malignant ECG variations. Tikkanen et al. classified ST segments as either horizontal/descending ( ≤ 0.1 mV within 100 ms after the J point) or upsloping/ascending (>0.1 mV elevation throughout the ST segment). The ascending early repolarization pattern was found to be more frequent (>85%) within the studied population of young athletes. In a large overall population, the horizontal/descending type was strongly associated with sudden cardiac death when compared to patients without early repolarization pattern, whereas the ascending pattern did not show significant association with sudden cardiac death (29, 30).

Roten et al. compared ECGs of healthy, asymptomatic patients with an early repolarization pattern ECG to patients with an early repolarization pattern ECG and history of ventricular fibrillation. The latter group had significantly longer QTc intervals, J waves and higher J wave amplitudes, low-amplitude T waves were found more frequently, and a lower T/R ratio. A low T/R ratio showed the strongest association with a malignant early repolarization variant among all studied parameters (31).

Bastiaenen et al. demonstrated the usefulness of exercise tolerance testing and Ajmaline provocation testing to unmask more malignant variants of early repolarization patterns. They conducted a study on 229 patients with a history of survived sudden cardiac death, ventricular arrhythmias, unexplained syncopes and a positive family history of sudden cardiac death. In 11% of studied patients, an early repolarization pattern was present in the baseline ECG. Exposing these patients with early repolarization pattern to exercise tolerance testing and Ajmaline provocation testing resulted in the disappearance of all ascending early repolarization patterns and patterns in the lateral leads. Sixty percent of horizontal/descending early repolarization patterns disappeared during Ajmaline provocation testing and only 25% disappeared during exercise tolerance testing. Patients with persistent early repolarization pattern showed a significantly higher frequency of arrhythmia related symptoms than patients in which early repolarization pattern had disappeared during testing (32–34).

Studies showed that patients with early repolarization pattern, found in the inferior leads, have a higher risk of mortality and sudden cardiac death. In patients aged older than 50 years, mortality between groups with and without early repolarization pattern additionally separates. Early repolarization may be interpreted to especially increase the risk of sudden cardiac death in the presence of additional triggers, such as acute ischemic events (10, 11, 13).

In asymptomatic patients, an electrophysiology study including programmed ventricular stimulation was not predictive for later ventricular arrhythmia in early repolarization syndrome patients (35). However in near future, advanced invasive and non-invasive electrophysiology technologies may improve risk stratification in patients with early repolarization pattern: Electrocardiographic imaging is a novel, non-invasive imaging technology which is based on a combination of cardiac electrical data recorded on the body surface and cardiac CT imaging. This technique can be used to create epicardial potential maps, and maps of epicardial activation and repolarization. Using this technology, Ghosh et al. recently demonstrated abnormally short activation-recovery intervals in inferior and lateral ventricular regions in patients with early repolarization syndrome, suggesting augmented repolarization and repolarization gradients in these areas that may represent substrates for ventricular arrhythmias. In the future, electrocardiographic imaging could prove to be a valuable tool for differentiating between malignant and benign forms of ER syndrome, thereby enhancing risk stratification (36).

Another novel technology that could potentially enhance risk stratification is a catheter equipped with monophasic action potential (MAP) electrodes. MAP catheters facilitate direct measurements of action potential characteristics and has the potential to characterize transmural repolarization gradients in detail. This may provide a more detailed characterization of the arrhythmogenic substrate in early repolarization syndrome patients. Invasive studies using this technique may be valuable in further investigating early repolarization patients who are at intermediate or high risk of sudden death (37).

## Therapy

As an early repolarization pattern is relatively frequent ECG-finding in the general population and the incidence of idiopathic ventricular fibrillation or polymorphic ventricular tachycardia is relatively low, the majority of individuals presenting an early repolarization pattern ECG will remain asymptomatic and the isolated presence of an early repolarization pattern does not require further intervention.

### ICD-implantation

Conversely, in patients with an early repolarization pattern who survived sudden cardiac death (early repolarization syndrome), the implantation of an implantable cardioverter defibrillator is indicated (27). The 2013 HRS/EHRA/APHRS Expert Consensus Statement gives recommendations for therapeutic interventions (Table 1):

**Table 1:** 2013 HRS/EHRA/APHRS Expert Consensus Statement recommendations for ICD implantation in early repolarization syndrome patients.

**Class I**	**1. ICD implantation is recommended in patients with a diagnosis of early repolarization syndrome who have survived a cardiac arrest**.
Class IIa	2. Isoproterenol infusion **can be useful** in suppressing electrical storms in patients with a diagnosis of early repolarization syndrome. 3. Quinidine in addition to an ICD **can be useful** for secondary prevention of VF in patients with a diagnosis of early repolarization syndrome.
Class IIb	4. ICD implantation **may be considered** in symptomatic family members of early repolarization syndrome patients with a history of syncope in the presence of ST segment elevation >1mm in 2 or more inferior or lateral leads. 5. ICD implantation **may be considered** in asymptomatic individuals who demonstrate a high-risk early repolarization ECG pattern (high J-wave amplitude, horizontal/descending ST-segment) in the presence of a strong family history of juvenile unexplained sudden death with or without a pathogenic mutation.
Class III	6. ICD implantation **is not recommended** in asymptomatic patients with an isolated early repolarization ECG pattern.

In the setting of survived sudden cardiac death, a familial screening is recommended. In a case of isolated, asymptomatic early repolarization ECG pattern, there is no recommendation for familial screening.

### Drug therapy

The incidence of ventricular fibrillation episodes and electrical storm is relatively common in early repolarization syndrome patients after ICD implantation. Regarding drug therapy in this situation, there is evidence that isoproterenol infusion acutely suppresses recurrent ventricular fibrillation in these patients. The dose can be initiated with 1.0 μg/min and should target a 20% in baseline heart rate or an absolute heart rate > 90 bpm, adapted to hemodynamic conditions and suppression of arrhythmia. The adrenergic activation with isoproterenol is likely effective by augmenting inward currents (particularly L-type Ca^2+^) which offset the net outward K^+^ current excess. Also the use of Quinidine or Hydroquinidine has been described to achieve a long-term suppression of ventricular fibrillation and ventricular tachycardia in early repolarization syndrome (38, 39). Quinidine, which inhibits outward currents, mainly I_to_, reduces the amplitude of the J wave and ST segment. The targeted range of Hydroquinidine serum levels should be 2–5 μg/ml, therefore a daily dose of 600 mg Hydroquinidine was applied in studies. In addition, Quinidine may reduce the early repolarization pattern or even restore a normal ECG in patients. The administration of Cilostazol, an oral phosphodiesterase III inhibitor which has been shown to be an effective drug therapy in Brugada syndrome, has also been described to successfully terminate ventricular fibrillation episodes, which were refractory to Quinidine therapy in early repolarization syndrome. Cilostazol has been shown to have a significant effect to block I_to_ and to augment I_Ca_, reducing the occurrence of phase 2 reentry phenomenon (40, 41).

## Conclusion

Whereas, an early repolarization pattern is frequently found in the overall population, the incidence of idiopathic ventricular fibrillation and the risk of early repolarization syndrome is relatively low. Several studies distinguished benign from more malignant early repolarization patterns. However, the mismatch between frequently found early repolarization ECG patterns, low incidences of early repolarization syndrome related sudden cardiac deaths, but fatal, grave consequences in affected patients remains a clinical challenge.

## Author contributions

FB, AD, GC, AL, KV, MT, TK, AF, JD, TP, NK, ND, FS, PJ, MiH and MéH Review of articles, preparation of manuscript, revision of article.

### Conflict of interest statement

The authors declare that the research was conducted in the absence of any commercial or financial relationships that could be construed as a potential conflict of interest.

## References

[1] Chugh SS Kelly KL Titus JL. Sudden cardiac death with apparently normal heart. Circulation (2000) 102:649–54. 10.1161/01.CIR.102.6.649 10931805

[2] Wever EF Robles de Medina EO. Sudden death in patients without structural heart disease. J Am Coll Cardiol. (2004) 43:1137–44. 10.1016/j.jacc.2003.10.053 15063419

[3] Shipley RA Hallaran WR The four-lead electrocardiogram in two hundred normal men and women. Am Heart J. (1936) 11:325–45. 10.1016/S0002-8703(36)90417-9

[4] Tomaszewski W Changement electrocardiographiques observes chez un homme mort de froid [Electrocardiographic changes observed in a man frozen to death]. Arch Mal Coeur. (1938) 31:525–8.

[5] Grant RP Estes HE Doyle JT. Spatial vector electrocardiography; the clinical characteristics of S-T and T vectors. Circulation (1951) 3:182–97. 10.1161/01.CIR.3.2.182 14812646

[6] Osborn JJ. Experimental hypothermia: respiratory and blood pH changes in relation to cardiac function. Am J Physiol. (1953) 175:389–98. 10.1152/ajplegacy.1953.175.3.389 13114420

[7] Derval N Shah A Jais P. Definition of early repolarization: a tug of war. Circulation (2011) 124:2185–6. 10.1161/CIRCULATIONAHA.111.064063 22083146

[8] Haïssaguerre M Derval N Sacher F Jesel L Deisenhofer I de Roy L. Sudden cardiac arrest associated with early repolarization. N Engl J Med. (2008) 358:2016–23. 10.1056/NEJMoa071968 18463377

[9] Jones RL Rubal B Jones S Steel K Davenport E Nasir J Prevalence of early repolarization in a large cohort of young adults. J Am Coll Cardiol. (2015) 65:A371 10.1016/S0735-1097(15)60371-0

[10] Haruta D Matsuo K Tsuneto A Ichimaru S Hida A Sera N. Incidence and prognostic value of early repolarization pattern in the 12-lead electrocardiogram. Circulation (2011) 123:2931–7. 10.1161/CIRCULATIONAHA.110.006460 21646495

[11] Sinner MF Reinhard W Müller M Beckmann BM Martens E Perz S. Association of early repolarization pattern on ECG with risk of cardiac and all-cause mortality: a population-based prospective cohort study (MONICA/KORA). PLoS Med. (2010) 7:e1000314. 10.1371/journal.pmed.1000314 20668657 PMC2910598

[12] Rosso R Kogan E Belhassen B Rozovski U Scheinman MM Zeltser D. J-point elevation in survivors of primary ventricular fibrillation and matched control subjects. J Am Coll Cardiol. (2008) 52:1231–8. 10.1016/j.jacc.2008.07.010 18926326

[13] Tikkanen JT Anttonen O Junttila MJ Aro AL Kerola T Rissanen HA. Long-term outcome associated with early repolarization on electrocardiography. N Engl J Med. (2009) 361:2529–37. 10.1056/NEJMoa0907589 19917913

[14] Benito B Guasch E Rivard L Nattel S. Clinical and mechanistic issues in early repolarization. J Am Coll Cardiol. (2010) 56:1177–786. 10.1016/j.jacc.2010.05.037 20883924

[15] Antzelevitch C Yan GX J wave syndromes. Heart Rhythm (2010) 7:549–58. 10.1016/j.hrthm.2009.12.006 20153265 PMC2843811

[16] Wu SH Lin XX Cheng YJ Qiang CC Zhang J. Early repolarization pattern and risk for arrhythmia death: a meta-analysis. J Am Coll Cardiol. (2013) 61:645–50. 10.1016/j.jacc.2012.11.023 23290543

[17] Liu XK Katchman A Whitfield BH Wan G Janowski EM Woosley RL Ebert SN. *In vivo* androgen treatment shortens the QT interval and increases the densities of inward and delayed rectifier potassium currents in orchiectomized male rabbits. Cardiovasc Res. (2003) 57:28–36. 10.1016/S0008-6363(02)00673-9 12504811

[18] Bai CX Kurokawa J Tamagawa M Nakaya H Furukawa T Non-transcriptional regulation of cardiac repolarization currents by testosterone. Circulation (2005) 112:1701–10. 10.1161/CIRCULATIONAHA.104.523217 16157773

[19] Nunn LM Bhar-Amato J Lowe MD Macfarlane PW Rogers P McKenna WJ. Prevalence of J-point elevation in sudden arrhythmic death syndrome families. J Am Coll Cardiol. (2011) 58:286–90. 10.1016/j.jacc.2011.03.028 21737021

[20] Noseworthy PA Tikkanen JT Porthan K Oikarinen L Pietilä A Harald K. The early repolarization pattern in the general population: clinical correlates and heritability. J Am Coll Cardiol. (2011) 57:2284–9. 10.1016/j.jacc.2011.04.003 21600720 PMC3183435

[21] Reinhard W Kaess BM Debiec R Nelson CP Stark K Tobin MD. Heritability of early repolarization: a population-based study. Circ Cardiovasc Genet. (2011) 4:134–8. 10.1161/CIRCGENETICS.110.958298 21282333

[22] Haïssaguerre M Chatel S Sacher F Weerasooriya R Probst V Loussouarn G. Ventricular fibrillation with prominent early repolarization associated with a rare variant of KCNJ8/KATP channel. J Cardiovasc Electrophysiol. (2009) 20:93–8. 10.1111/j.1540-8167.2008.01326.x 19120683

[23] Medeiros-Domingo A Tan BH Crotti L Tester DJ Eckhardt L Cuoretti A. Gain-of-function mutation S422L in the KCNJ8-encoded cardiac K(ATP) channel Kir6.1 as a pathogenic substrate for J-wave syndromes. Heart Rhythm (2010) 7:1466–71. 10.1016/j.hrthm.2010.06.016 20558321 PMC3049900

[24] Burashnikov E Pfeiffer R Barajas-Martinez H Delpón E Hu D Desai M. Mutations in the cardiac L-type calcium channel associated with inherited J-wave syndromes and sudden cardiac death. Heart Rhythm (2010) 7:1872–82. 10.1016/j.hrthm.2010.08.026 20817017 PMC2999985

[25] Watanabe H Nogami A Ohkubo K Kawata H Hayashi Y Ishikawa T. Electrocardiographic characteristics and SCN5A mutations in idiopathic ventricular fibrillation associated with early repolarization. Circ Arrhythm Electrophysiol. (2011) 4:874–81. 10.1161/CIRCEP.111.963983 22028457

[26] Macfarlane PW Antzelevitch C Haissaguerre M Huikuri HV Potse M Rosso R. The early repolarization pattern. J Am Coll Cardiol. (2015) 66:470–77. 10.1016/j.jacc.2015.05.033 26205599

[27] Priori SG Wilde AA Horie M Cho Y Behr ER Berul C. HRS/EHRA/APHRS expert consensus statement on the diagnosis and management of patients with inherited primary arrhythmia syndromes: document endorsed by HRS, EHRA, and APHRS in May 2013 and ACCF, AHA, PACES, and AEPC in June 2013. Heart Rhythm (2013) 10:1932–63. 10.1016/j.hrthm.2013.05.014 24011539

[28] Nam GB Ko KH Kim J Park KM Rhee KS Choi KJ. Mode of onset of ventricular fibrillation in patients with early repolarization pattern vs. Brugada syndrome. Eur Heart J. (2010) 31:330–9. 10.1093/eurheartj/ehp423 19880418 PMC2814221

[29] Tikkanen JT Junttila MJ Anttonen O Aro AL Luttinen S Kerola T. Early repolarization: electrocardiographic phenotypes associated with favorable long-term outcome. Circulation (2011) 123:2666–73. 10.1161/CIRCULATIONAHA.110.014068 21632493

[30] Viskin S Havakuk O Antzelevitch C Rosso R. Malignant early repolarization: it's the T-wave, stupid. Heart Rhythm (2016) 13:903–4. 10.1016/j.hrthm.2015.12.017 26690063

[31] Roten L Derval N Maury P Mahida S Pascale P Leenhardt A. Benign vs malignant inferolateral early repolarization: focus on the T wave. Heart Rhythm (2016) 13:894–902. 10.1016/j.hrthm.2015.11.020 26592849

[32] Rautaharju PM Surawicz B Gettes LS Bailey JJ Childers R Deal BJ. AHA/ACCF/HRS recommendations for the standardization and interpretation of the electrocardiogram: part IV: the ST segment, T and U waves, and the QT interval: a scientific statement from the American Heart Association Electrocardiography and Arrhythmias Committee, Council on Clinical Cardiology; the American College of Cardiology Foundation; and the Heart Rhythm Society: endorsed by the international society for computerized electrocardiology. J Am Coll Cardiol. (2009) 53:982–91. 10.1016/j.jacc.2008.12.014 19281931

[33] Bastiaenen R Raju H Sharma S Papadakis M Chandra N Muggenthaler M. Characterization of early repolarization during ajmaline provocation and exercise tolerance testing. Heart Rhythm (2013) 10:247–54. 10.1016/j.hrthm.2012.10.032 23089898

[34] Refaat MM Hotait M Tseng ZH. Utility of the exercise electrocardiogram testing in sudden cardiac death risk stratification. Ann Noninvasive Electrocardiol. (2014) 19:311–8. 10.1111/anec.12191 25040480 PMC6932419

[35] Mahida S Derval N Sacher F Leenhardt A Deisenhofer I Babuty D. Role of electrophysiological studies in predicting risk of ventricular arrhythmia in early repolarization syndrome. J Am Coll Cardiol. (2015) 65:151–9. 10.1016/j.jacc.2014.10.043 25593056

[36] Ghosh S Cooper DH Vijayakumar R Zhang J Pollak S Haissaguerre M. Early repolarization associated with sudden death: insights from noninvasive electrocardiographic imaging. Heart Rhythm (2010) 7:534–7. 10.1016/j.hrthm.2009.12.005 20153422 PMC2865425

[37] Mahida S Derval N Sacher F Berte B Yamashita S Hooks DA. History and clinical significance of early repolarization syndrome. Heart Rhythm (2015) 12:242–9. 10.1016/j.hrthm.2014.09.048 25257090

[38] Nam GB Kim YH Antzelevitch C. Augmentation of J waves and electrical storms in patients with early repolarization. N Engl J Med. (2008) 358:2078–9. 10.1056/NEJMc0708182 18463391 PMC2515862

[39] Haïssaguerre M Sacher F Nogami A Komiya N Bernard A Probst V. Characteristics of recurrent ventricular fibrillation associated with inferolateral early repolarization role of drug therapy. J Am Coll Cardiol. (2009) 53:612–9. 10.1016/j.jacc.2008.10.044 19215837

[40] Iguchi K Noda T Kamakura S Shimizu W. Beneficial effects of cilostazol in a patient with recurrent ventricular fibrillation associated with early repolarization syndrome. Heart Rhythm (2013) 10:604–6. 10.1016/j.hrthm.2012.11.001 23142636

[41] Patocskai B Barajas-Martinez H Hu D Gurabi Z Koncz I Antzelevitch C. Cellular and ionic mechanisms underlying the effects of cilostazol, milrinone, and isoproterenol to suppress arrhythmogenesis in an experimental model of early repolarization syndrome. Heart Rhythm (2016) 13:1326–34. 10.1016/j.hrthm.2016.01.024 26820510 PMC4879023

